# Impact of mechanical bowel preparation on the gut microbiome of patients undergoing left-sided colorectal cancer surgery: randomized clinical trial

**DOI:** 10.1093/bjs/znae213

**Published:** 2024-09-02

**Authors:** Kristina Žukauskaitė, Angela Horvath, Žilvinas Gricius, Mindaugas Kvietkauskas, Bernardas Baušys, Audrius Dulskas, Justas Kuliavas, Rimantas Baušys, Simona Rūta Letautienė, Ieva Vaicekauskaitė, Rasa Sabaliauskaitė, Augustinas Baušys, Vanessa Stadlbauer, Sonata Jarmalaitė

**Affiliations:** Institute of Biosciences, Life Sciences Centre, Vilnius University, Vilnius, Lithuania; Department of Gastroenterology and Hepatology, Medical University of Graz, Graz, Austria; Department of Gastroenterology and Hepatology, Medical University of Graz, Graz, Austria; Centre for Biomarker Research in Medicine (CBmed GmbH), Graz, Austria; Clinic of Gastroenterology, Nephrourology, and Surgery, Institute of Clinical Medicine, Faculty of Medicine, Vilnius University, Vilnius, Lithuania; Clinic of Gastroenterology, Nephrourology, and Surgery, Institute of Clinical Medicine, Faculty of Medicine, Vilnius University, Vilnius, Lithuania; Clinic of Gastroenterology, Nephrourology, and Surgery, Institute of Clinical Medicine, Faculty of Medicine, Vilnius University, Vilnius, Lithuania; Clinic of Gastroenterology, Nephrourology, and Surgery, Institute of Clinical Medicine, Faculty of Medicine, Vilnius University, Vilnius, Lithuania; National Cancer Institute, Vilnius, Lithuania; Clinic of Gastroenterology, Nephrourology, and Surgery, Institute of Clinical Medicine, Faculty of Medicine, Vilnius University, Vilnius, Lithuania; National Cancer Institute, Vilnius, Lithuania; National Cancer Institute, Vilnius, Lithuania; National Cancer Institute, Vilnius, Lithuania; Institute of Biosciences, Life Sciences Centre, Vilnius University, Vilnius, Lithuania; National Cancer Institute, Vilnius, Lithuania; Institute of Biosciences, Life Sciences Centre, Vilnius University, Vilnius, Lithuania; National Cancer Institute, Vilnius, Lithuania; Institute of Biosciences, Life Sciences Centre, Vilnius University, Vilnius, Lithuania; National Cancer Institute, Vilnius, Lithuania; Department of Pathology and Forensic Medicine, Faculty of Medicine, Institute of Biomedical Sciences, Vilnius University, Vilnius, Lithuania; Department of Gastroenterology and Hepatology, Medical University of Graz, Graz, Austria; Centre for Biomarker Research in Medicine (CBmed GmbH), Graz, Austria; Institute of Biosciences, Life Sciences Centre, Vilnius University, Vilnius, Lithuania; National Cancer Institute, Vilnius, Lithuania

## Abstract

**Background:**

Postoperative complications after colorectal cancer surgery have been linked to the gut microbiome. However, the impact of mechanical bowel preparation using oral preparation agents or rectal enema on postoperative infections remains poorly understood. This study aimed to compare the impact of oral preparation and rectal enema on the gut microbiome and postoperative complications.

**Methods:**

This open-label pilot RCT was conducted at the National Cancer Institute, Vilnius, Lithuania. Patients with left-side colorectal cancer scheduled for elective resection with primary anastomosis were randomized 1 : 1 to preoperative mechanical bowel preparation with either oral preparation or rectal enema. Stool samples were collected before surgery, and on postoperative day 6 and 30 for 16S rRNA gene sequencing analysis. The primary outcome was difference in β-diversity between groups on postoperative day 6.

**Results:**

Forty participants were randomized to oral preparation (20) or rectal enema (20). The two groups had similar changes in microbiome composition, and there was no difference in β-diversity on postoperative day 6. Postoperative infections occurred in 12 patients (32%), without differences between the study groups. Patients with infections had an increased abundance of bacteria from the Actinomycetaceae family, *Actinomyces* genus, *Sutterella* uncultured species, and *Enterococcus faecalis* species.

**Conclusion:**

Mechanical bowel preparation with oral preparation or rectal enema resulted in similar dysbiosis. Patients who experienced postoperative infections exhibited distinct gut microbiome compositions on postoperative day 6, characterized by an increased abundance of bacteria from the Actinomycetaceae family, *Actinomyces* genus, *Sutterella* uncultured species, and *Enterococcus faecalis* species.

**Registration number:**

NCT04013841 (http://www.clinicaltrials.gov).

## Teaser

Postoperative complications after colorectal cancer surgery have been linked to the gut microbiome, but the impact of mechanical bowel preparation (MBP) using oral preparation agents (OP) or rectal enema (RE), on postoperative infections remains poorly understood. This study aimed to compare the impact of OP and RE on the gut microbiome and postoperative complications. MBP with OP and RE resulted in similar dysbiosis. Patients who experienced postoperative infections exhibited distinct gut microbiome compositions on postoperative day 6, characterized by an increased abundance of bacteria from the Actinomycetaceae family, *Actinomyces* genus, *Sutterella* uncultured species, and *Enterococcus faecalis* species.

## Introduction

The use of mechanical bowel preparation (MBP) in colorectal cancer surgery is a subject of ongoing discussion. Presently, there is no robust evidence to strongly endorse the clinical advantages of MBP when administered alongside conventional systemic antibiotic prophylaxis for colonic surgery. Nevertheless, its use is recommended in rectal surgery, partly supported by results from the GRECCAR III and MOBILE2 RCTs. The GRECCAR III study^1^ revealed that MBP notably diminished the incidence of postoperative complications, particularly infections, whereas MOBILE2^2^ showed decreased morbidity when MBP was combined with oral antibiotics. Many surgeons prefer MBP because of its technical advantages, including easier bowel handling, improved ability to detect small tumours and polyps, and facilitation of on-table endoscopy^3^. It is important to acknowledge that most MBP-related evidence stems from studies testing osmotic diarrhoea-inducing oral preparation (OP) agents. Despite the benefits, OP has notable adverse effects, such as vomiting, abdominal pain, hypovolaemia, and metabolic disturbances^1,4^. Moreover, it triggers modifications and inflammatory responses in the colon^5^, along with alterations in the colonic microbiome similar to those observed in other gastrointestinal conditions such as inflammatory bowel disease^6^. Both inflammatory changes in the bowel wall and gut microbiome alterations could potentially contribute to impaired anastomotic healing, thus increasing the risk of anastomotic insufficiency^5,7^. Moreover, these factors might also contribute to other postoperative complications, such as pneumonia, wound infection, intra-abdominal abscess, and urinary tract infection^8,9^.

Rectal enema (RE) presents an alternative approach to OP, but its adoption among colorectal surgeons is notably less common. Pittet *et al.*^10^ demonstrated that RE can attain efficacy of bowel preparation comparable to that of OP in the context of rectal cancer surgery. The advantages and disadvantages of RE for MBP preceding colorectal cancer surgery have not been explored comprehensively. Uncertainty remains regarding whether RE has a similarly significant impact on the gut microbiome as OP. Thus, this pilot RCT was designed to compare OP with RE for patients undergoing left-side colorectal cancer resection, specifically examining the impact on the gut microbiome and its role in postoperative complications.

## Methods

### Study design and ethics

This pilot two-arm RCT was conducted at the National Cancer Institute, Vilnius, Lithuania, after the protocol had been approved by the Vilnius Regional Biomedical Research Ethics Committee (2019/6-1133-631) and registered at ClinicalTrials.gov (NCT04013841). The study was conducted in accordance with the ethical standards of the Helsinki Declaration of 1975, as revised in 2013. All patients provided written informed consent before participating in the study.

### Study participants

Patients aged over 18 years with histologically confirmed or clinically suspected left-sided colorectal cancer scheduled for elective surgery were eligible for inclusion. Patients were excluded if they: had surgery with ileostomy planned; had a history of allergy to OP agents; required multivisceral resection; had emergency surgery; had a history of inflammatory bowel disease; had a history of gastrointestinal surgery; had clinical signs of bowel obstruction that would contraindicate OP; or were pregnant. Participants were screened for eligibility by the multidisciplinary team and informed about the study by one of the investigators at the outpatient visit. Those who were willing to participate were randomized.

### Randomization

Participants were randomized to receive either OP or RE in a 1 : 1 allocation according to a computer-generated randomization schedule. The randomization sequence was created using a freely available online tool (https://www.sealedenvelope.com/), with only the principal investigator having access to the randomization sequence. Random allocations were numbered sequentially in sealed opaque envelopes by assisting personnel who were not subsequently involved in the study, and opened the day before the surgery. Owing to the nature of the intervention, neither the participants nor investigators were blinded; however, data collection and analyses were subsequently performed blindly.

### Preoperative bowel preparation and treatment

For preoperative MBP, patients in the OP group received 4 litres of the oral agent macrogol 4000 (73.69 mg/1; Fortrans^®^; Ipsen Pharma, Paris, France) starting the afternoon before surgery. RE was administered as 2 litres of 0.9% sodium chloride via an irrigator (Plasti-med, Istanbul, Turkey) the evening before surgery. All patients were given antibiotic prophylaxis before surgery; a single dose of intravenous cefazolin 2 g and metronidazole 500 mg were administered 30–60 min before incision. This was a single-centre study, with all participants undergoing standardized colorectal resection according to the institutional protocol at the National Cancer Institute (Vilnius, Lithuania), via either an open or laparoscopic approach. The postoperative care pathway adhered to enhanced recovery after surgery principles^11^.

### Study outcomes

The primary outcome of the study was the difference in β-diversity between OP and RE groups on postoperative day (POD) 6. Secondary outcomes were: difference in β-diversity between OP and RE groups on POD30 ; difference in α-diversity between OP and RE groups on POD6 and POD30; 30-day morbidity rate; and quality of MBP as assessed during surgery by a surgeon on a Likert scale from 1 (very poor) to 10 (excellent).

### Stool sample collection and sequencing

Fresh stool samples were collected from study participants and immediately stored at −80°C at baseline (1 day before bowel preparation), and on POD6 and POD30. After all samples had been collected, frozen samples were shipped on dry ice to the Medical University of Graz (Graz, Austria). DNA was extracted using a MagNA Pure LC DNA Isolation Kit III (Bacteria, Fungi) (Roche, Mannheim, Germany) according to the manufacturer’s recommendations. Hypervariable region V1–V2 was amplified (primers: F-AGAGTTTGATCCTGGCTCAG; R-CTGCTGCCTYCCGTA) and 16S rRNA gene sequencing was performed using Illumina Miseq technology (Illumina, Eindhoven, the Netherlands), as reported previously^12^.

### Processing of sequencing data

Raw sequencing data were processed using QIIME 2 tools on a local Galaxy instance^13^. Denoising was done with DADA2^14^. To ensure the integrity of the sequencing data, read sequences were truncated at 250 and 200 bases for forward and reverse reads respectively. After processing, filtering, and rarefying the sequencing data, a total of 2 180 886 sequencing reads remained. On average, each sample had 22 483 reads available for further analysis, with a minimum of 3348 and a maximum of 31 882 reads. Taxonomy was assigned based on the Silva 132 database release at 99% amplicon sequence variant (ASV) level with a naive Bayes classifier. Additionally, sensitivity analysis was performed with higher rarefaction depth (8444), excluding two samples with low sequencing depth. ASVs that were abundant in negative sequencing controls and *Cyanobacteria* were removed from further analysis as potential contaminants.

### Statistical analysis

The α-diversity was quantified in terms of the richness, Shannon index, inverse Simpson, and evenness indices. The β-diversity was examined by principal coordinate analysis based on a Bray–Curtis’ dissimilarity matrix, with results evaluated using permutational multivariate ANOVA using R version 4.3.0 (R Core Team, Vienna, Austria)^15^. Subsequently, linear discriminant analysis effect-size (LEfSe) analysis was undertaken to identify features that exhibited differential abundance between different MBP groups to determine their effect sizes using the microbiomeMarker package^16^. A linear discriminant analysis cut-off value of 3 was applied uniformly to both groups, enabling the identification of substantial differences in abundance with increased precision. Subsequently, a linear model was employed to ascertain the statistical significance of the results obtained using the lme4 package^17,18^. Figures were created using the ggplotify package^19^.

Statistical analysis of clinical variables was carried out using SPSS^®^ version 29.0.1.0 (IBM, Armonk, NY, USA). The Shapiro–Wilk test was used to test normality. Categorical variables were compared using the χ^2^ test or Fisher’s exact test, and the Mann–Whitney *U* test was used for continuous variables. Spearman’s rank correlation coefficient was employed to investigate relationships between the variables. The log rank test was used to evaluate differences in the probability of infection between groups. *P* < 0.050 was taken as the threshold for statistical significance.

In the absence of similar previous studies and inability to assume effect size, it was not feasible to conduct formal sample size calculations for this study describing microbiome composition in terms of β-diversity. Consequently, 20 participants were recruited to each group as an adequate number to detect medium-to-large effect sizes^20^.

## Results

### Baseline characteristics

**Fig. 1:**
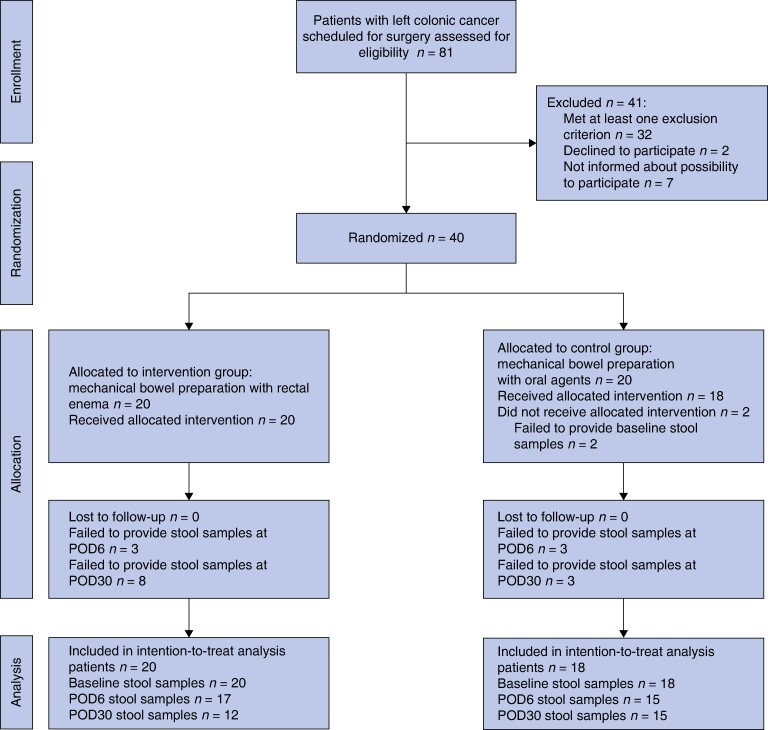
CONSORT diagram for the study

Between April and November 2021, 40 participants were randomized to OP (20) or RE (20) groups. After allocation, two patients in the OP group were excluded because of their inability to provide stool samples on either POD6 or POD30 (*Fig. 1*).

The OP and RE groups were well balanced in terms of demographic, clinicopathological, and treatment characteristics, except that patients in the OP group were younger (median 61 (i.q.r. 53–67) *versus* 71 (65–77) years; *P* = 0.012) (*Table 1*). There were no adverse events related to the bowel preparation.

**Table 1:** Baseline and treatment characteristics

	Rectal enema (*n* = 20)	Oral preparation (*n* = 18)
**Age (years), median (i.q.r.)**	71 (65–77)	61 (53–67)
**Sex**		
Male	12	8
Female	8	10
**BMI (kg/m^2^), median (i.q.r.)**	27.9 (24.7–30.6)	28.5 (27.4–29.5)
**Active smoker**	1 (5%)	0 (0%)
**Charlson Co-morbidity Index score**		
> 5	7 (35%)	3 (17%)
≤ 5	13 (65%)	15 (83%)
**Tumour location**		
Sigmoid or descending colon	9 (45%)	12 (67%)
Rectum	11 (55%)	6 (33%)
**Histology**		
Adenocarcinoma	18 (90%)	17 (94%)
Non-cancerous lesions	2 (10%)	1 (6%)
**Disease stage**		
I–II	8 (40%)	13 (72%)
III–IV	10 (50%)	4 (22%)
Not applicable	2 (10%)	1 (6%)
**Type of surgery**		
Left colectomy or sigmoidectomy	9 (45%)	12 (67%)
Rectal resection	11 (55%)	6 (33%)
**Surgical approach**		
Laparoscopic	12 (60%)	14 (78%)
Open	8 (40%)	4 (22%)
**Duration of surgery (min), median (i.q.r.)**	140 (101–153)	112 (95–147)
**Duration of hospital stay (days), median (i.q.r.)**	9 (8–14)	9 (6–11)
**History of antibiotic, probiotic, prebiotic use within 1 month before enrolment**	0 (0)	0 (0)
**Neoadjuvant therapy**	0 (0)	0 (0)

### Primary outcome: impact of bowel preparation on β-diversity of intestinal microbiome on POD6

**Fig. 2:**
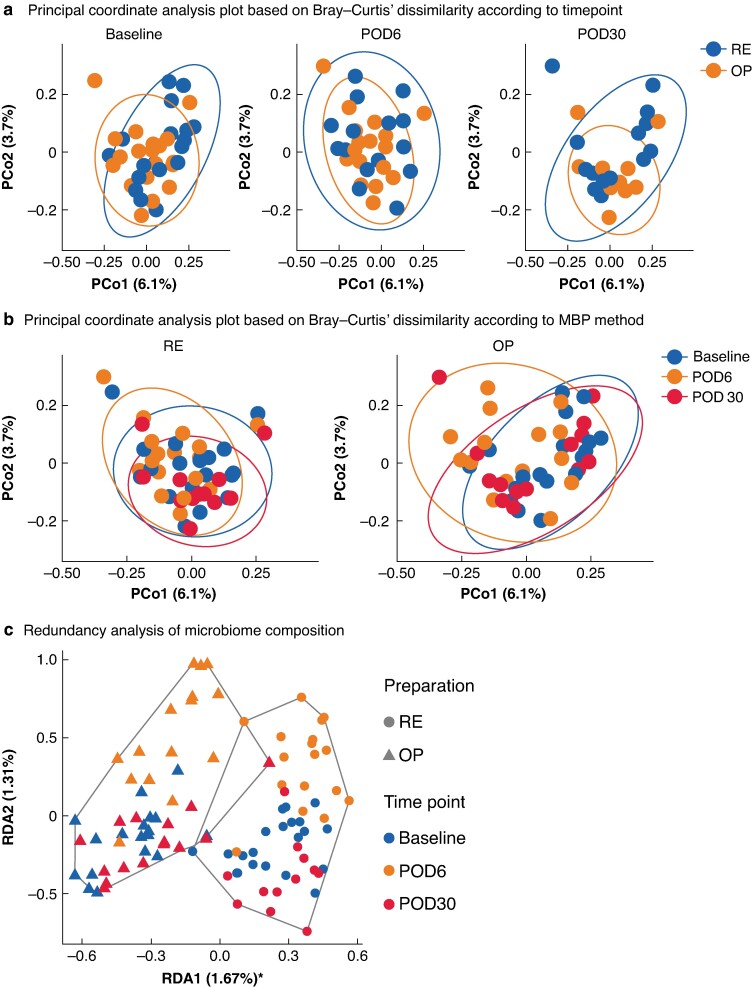
Principal coordinate analysis plot based on Bray–Curtis’ dissimilarity and Redundancy analysis of microbiome composition **a**,**b** PCo1 and PCo2 correspond to the first and second principal coordinates, derived from the Bray–Curtis dissimilarity matrix. These axes capture major variations in microbiome composition between samples. **c** RDA1 and RDA2 represent the first and second axes of RDA, summarizing the relationships between groups and microbial community composition. POD, postoperative day; RE, rectal enema; OP, oral preparation; *The significance of RDA1 suggests that this axis explains a substantial proportion of the variance in the data, indicating that the explanatory variables associated with RDA1 are highly relevant in describing the patterns observed in the response variables.

Baseline β-diversity was comparable between the study groups (*P* = 0.226). Primary outcome analysis showed no difference in β-diversity on POD6 between the study groups (*P* = 0.198), nor on POD30 (*P* = 0.310) (*Fig. 2a*).

Despite the lack of differences between the study groups, both interventions for MBP (OP or RE) followed by colorectal resection resulted in significant alterations in gut microbiome composition at POD6 compared with baseline (*P* = 0.001). These differences remained significant 30 days after surgery (*P* = 0.001) (*Fig. 2b*). Comparison of POD6 and POD30 also showed significant differences (*P* = 0.002). Visual examination of the graphs in *Fig. 2* alongside the accompanying statistical data revealed that the MBP-induced changes in microbiome composition started to recover over time, but this recovery process may take longer than 30 days.

### Microbiome composition in rectal enema and oral preparation groups

**Fig. 3:**
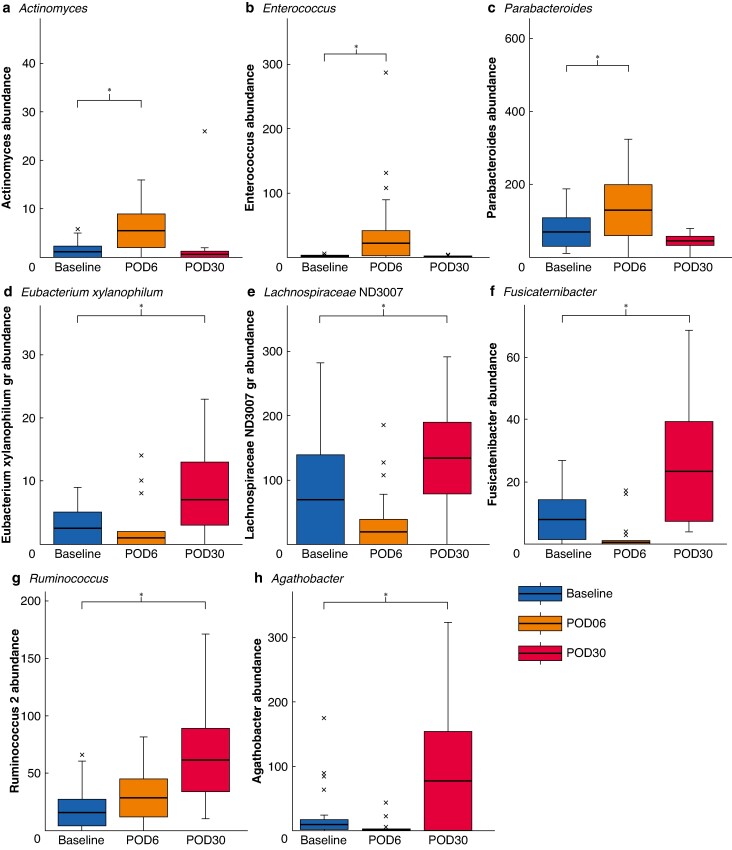
Comparative assessment of genera abundance in rectal enema group between different time points (compared with baseline) using linear discriminant analysis: effect-size model analysis Distribution of selected genera in rectal enema group throughout the 30-day follow-up: **a**  *Actinomyces*, **b**  *Enterococcus*, **c**  *Parabacteroides*, **d**  *Eubacterium xylanophylum*, **e***Lachnospiraceae* ND3007, **f**  *Fusicatenibacter*, **g**  *Ruminococcus* 2, and **h**  *Agathobacter*. Bold lines, boxes, and error bars indicate median, i.q.r., and range respectively. Crosses indicate outliers. POD, postoperative day. A generalized linear model was employed to ascertain the statistical significance of the results. *P < 0.050 (ANOVA test).

The LEfSe analysis, coupled with linear model analysis, unveiled significant shifts in the microbiome throughout the study in both groups. In the case of MBP with RE followed by resection, there was an increase in the abundance of bacteria from the *Actinomyces* genus, *Enterococcus* genus, *Parabacteroides* genus, and *Ruminococcus* 2 genus in the short term (POD6 *versus* baseline). However, most of these changes reverted to baseline by POD30, apart from those for the *Ruminococcus* 2 genus, which exhibited further increased abundance (*Fig. 3*).

**Fig. 4:**
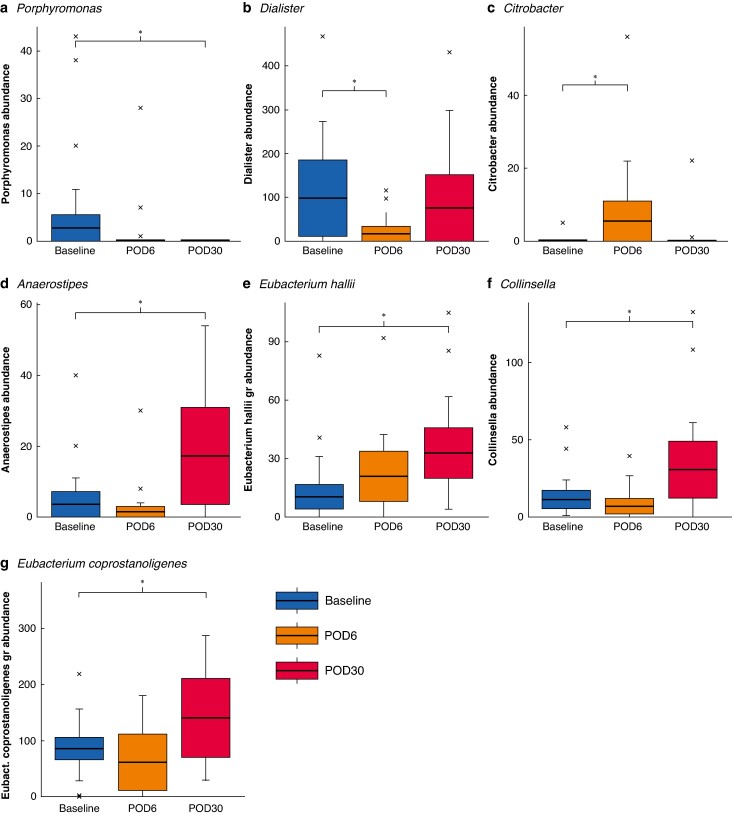
Comparative assessment of genera abundance in oral preparation group between different time points (compared with baseline) using linear discriminant analysis: effect-size model analysis Distribution of selected genera in oral preparation group throughout the 30-day follow-up: **a**  *Porphyromonoas*, **b**  *Dialister*, **c**  *Citrobacter*, **d**  *Anaerostipes*, **e**  *Eubacterium hallii*, **f**  *Collinsella*, and **g**  *Eubacterium coprostanoligenes*. Bold lines, boxes, and error bars indicate median, i.q.r., and range respectively. Crosses indicate outliers. POD, postoperative day. A generalized linear model was employed to ascertain the statistical significance of the results. *P < 0.050 (ANOVA test).

In the OP group, interventions led to a temporary decrease in *Dialister* and increase in *Citrobacter* genus abundance, observed on POD6, which returned to baseline levels by POD30. Persistent changes detected on both POD6 and POD30 included a decrease in bacteria from the *Porphyromonas* genus. Moreover, the prolonged impact of OP featured an increased abundance of *Eubacterium coprostanoligenes* genus, *Eubacterium hallii* genus group, and *Collinsella* genus (*Fig. 4*).

### Secondary outcomes: effect of bowel preparation method on α-diversity of intestinal microbiome

**Fig. 5:**
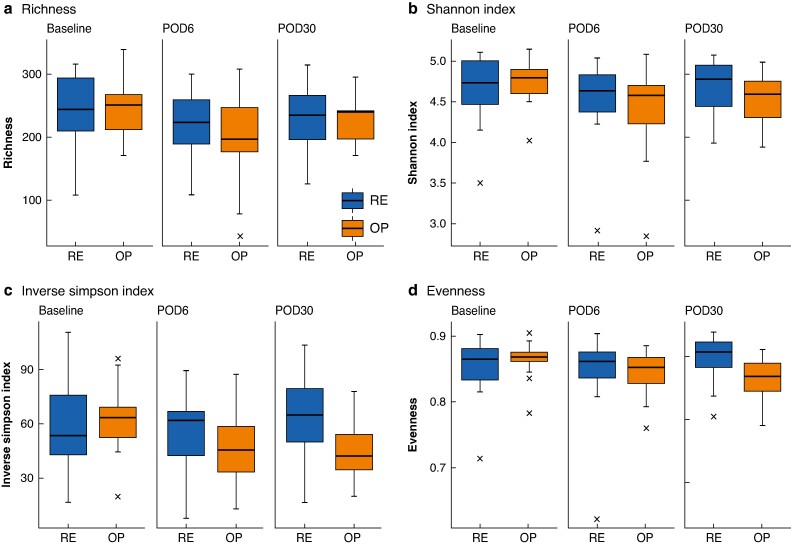
Changes in α-diversity parameters in patients undergoing mechanical bowel preparation before left-side colorectal resection **a** Richness, **b** Shannon index, **c** Inverse Simpson index, and **d** Evenness. Bold lines, boxes, and error bars indicate median, i.q.r., and range respectively. Crosses indicate outliers. POD, postoperative day; RE, rectal enema; OP, oral preparation.

There was no significant difference in α-diversity between the OP and RE groups. The α-diversity decreased slightly on POD6 and POD30 in the OP group, but not in the RE group. The most notable alterations were observed in the evenness and inverse Simpson index (*Fig. 5* and *Table S1*).

### Impact of bowel preparation method on postoperative outcomes

The method of MBP had no impact on the bowel preparation score (median 8 (i.q.r. 7–9) for RE *versus* 8 (7–9) for OP; *P* = 0.806). After colorectal cancer resection, 15 of 38 patients (39%) developed postoperative complications; 12 (32%) had infections and 3 (8%) developed ileus. Postoperative morbidity rates and severity of complications were similar in the two study groups (*Table 2*). Infections (wound infection, urinary tract infection, anastomotic leak, and intra-abdominal abscess) were the most common types of complication in both groups (RE 30% *versus* OP 33%; *P* = 0.825).

**Table 2:** Intraoperative and postoperative outcomes

	Rectal enema (*n* = 20)	Oral preparation agents (*n* = 18)	*P* *
**Patients with postoperative complications**	8 (40%)	7 (39%)	0.999
**Clavien–Dindo grade**			0.421
I–II	4 (20%)	6 (33%)	
III–IV	4 (20%)	1 (6%)	
**Type of complication**			
Wound infection	2 (10%)	4 (22%)	0.395
Urinary tract infection	1 (5%)	2 (11%)	0.595
Anastomotic leak	1 (5%)	0 (0%)	0.999
Intra-abdominal abscess	2 (10%)	0 (0%)	0.488
Ileus	3 (15%)	0 (0%)	0.232
Other	1 (5%)	3 (17%)	0.328

^*^χ^2^ test or Fisher’s exact test.

### *Post hoc* analysis: association between gut microbiome changes and postoperative infections

The association between changes in the gut microbiome and postoperative infections was investigated in a *post hoc* analysis. Patients who developed postoperative infections (irrespective of method of MBP) showed a higher abundance of bacteria from the Pseudomonadales order, Actinomycetales order, Actinobacteria class, Actinomycetaceae family, *Actinomyces* genus, *Sutterella* uncultured species, and *Enterococcus faecalis* species on POD6 compared with patients who did not develop a postoperative infection (*Fig. S1*).

The abundance of *E. faecalis* increased on POD6 and returned to baseline values by POD30 in both groups, with a steeper increase in the OP group (*P* < 0.001) (*Figs S2*, *S3* and *Table S2*). It is noteworthy that in two patients who experienced culture-confirmed postoperative infections caused by *E. faecalis*, there was a concurrent increase in the abundance of *E. faecalis* in the gut microbiome (*Table S3*).

## Discussion

This pilot RCT aimed to compare two different methods of MBP (OP and RE) with respect to the gut microbiome and its potential role in postoperative complications among patients undergoing left-side colorectal resection. The main findings were that both OP and RE led to similar and transient dysbiosis and outcomes after surgery. Additionally, the study identified specific gut microbiome changes in patients who experienced postoperative infections, including an increased abundance of bacteria from the Pseudomonadales order, Actinomycetales order, Actinobacteria class, Actinomycetaceae family, *Actinomyces* genus, *Sutterella* uncultured species, and *Enterococcus faecalis* species on POD6.

Recently, there has been a growing interest in exploring the potential impact of MBP on the composition of the gut microbiome; however, the existing body of evidence has yielded conflicting results. Numerous studies^21–25^ examining the composition of the gut microbiome after bowel cleansing with oral agents for colonoscopy have demonstrated disruptions in microbial composition shortly after the procedure. It is known that probiotic bacteria—a group of beneficial bacteria crucial for gut health—can have a positive impact on side-effects after screening colonoscopy^26^. Notably, the study by Drago *et al*.^25^ suggested that oral agents induce dysbiosis, characterised by a reduction in the abundance of typical probiotic Lactobacillaceae, which persists for at least a month. In contrast to these findings, most studies indicated that the composition of the gut microbiome returns to a baseline state within a considerably shorter time, typically around 14 days^22,24^. The present study has shown that MBP and colorectal resection has a significant impact on composition of the microbiome throughout the 30-day time frame.

This study is the first to reveal that RE induces a dysbiotic state comparable to that triggered by OP. However, it is important to note that, in this study, MBP was combined with surgery and that alterations in the gut microbiome may have resulted not only from MBP but also from the surgical procedure itself^27,28^. Surgical stress can induce alterations in the host that influence the intestinal microenvironment, ultimately causing dysbiosis^29^. It was shown that the composition and diversity of the gut microbiome of patients with colorectal cancer 1 month after surgery were significantly different from those of patients before operation and those from healthy individuals^27^. Another study^28^ evaluated the gut microbiota of patients who underwent surgical resection of colorectal cancer to investigate whether surgical treatment altered the microbial community; it was found that the gut microbiome differed between patients before and after operation. The relative abundance of phylum Proteobacteria increased after surgery (*Fig. S4*). An imbalance in gut microbiota frequently occurs owing to a persistent rise in Proteobacteria, a phylum that typically constitutes only a small portion of the human gut microbiota^27,30^. The present study was not designed to distinguish whether the increase in Proteobacteria abundance on POD6 was a result of the MBP, surgical intervention, or a combination of both.

Dysbiosis in surgical patients holds potential clinical significance as it may be associated with the development of postoperative complications, particularly postoperative infections^8,31^. It is well established that infections are the most common type of complication after colorectal cancer surgery^32,33^. This was corroborated here, where infections were frequent, occurring in approximately one-third of participants. Traditionally, it was believed that most surgical-site infections (SSIs) after elective surgery resulted from intraoperative contamination, although solid evidence supporting this notion has been lacking^31^. An alternative hypothesis, known as the Trojan Horse theory, posits that pathogens can be transported to the site of infection from distant locations by immune cells^34^. To bolster this hypothesis, it was demonstrated, using a clinically relevant experimental animal model, that the infecting organisms responsible for SSIs originated in the gastrointestinal tract^35^. The present findings indirectly support this theory by revealing distinct gut microbiome compositions in patients who developed postoperative infections after surgery compared with those who did not. Patients with infections exhibited an increased abundance of the Actinomycetaceae family, *Actinomyces* genus, *Sutterella* uncultured species, and *E. faecalis* species in samples from POD6. Intriguingly, among the four patients with available infection-site culture reports, two had infections caused by *E. faecalis* and both had an increased abundance of this bacterium in their gut. It is known that enterococci are opportunistic bacteria that can become pathogenic when they establish themselves in environments where they are not typically found. This group of bacteria is frequently encountered in patients who have acquired infections within hospital settings^36–38^, most of which are attributed to *E. faecalis*, which is 1 of over 20 different species within the *Enterococcus* genus^39,40^. In the context of colorectal surgery, *E. faecalis* may play a particularly perilous role as it has been linked to anastomotic leak^41^. The precise mechanism by which *E. faecalis* contributes to the pathogenesis of anastomotic leak is not fully understood at this time, but its collagenolytic activity, which can degrade anastomotic integrity, is a plausible contributing factor^41^. The role of other bacteria such as the *Actinomyces* and *Sutterella*, whose abundance was observed to increase in patients with postoperative infections, remains poorly understood and requires further investigation.

The present study has noteworthy limitations. First, surprisingly, the baseline microbiome composition exhibited disparities between the RE and OP groups, even though randomization was rigorously employed. Although a definitive explanation for this finding remains elusive, disparities in age between the study groups were potentially a contributing factor (*Fig. S5*). Previous research indicated that microbial stability and diversity tend to decrease as individuals age^42,43^. Such imbalances are not uncommon in pilot studies characterized by relatively small sample sizes, as is the present study. Second, the authors’ ability to investigate whether the microbes that exhibited enrichment in the gut microbiome on POD6 were present at the infection site was hindered by the absence of samples collected from the infection site for sequencing. Furthermore, culture reports were accessible for only a limited number of patients with infections. Third, in the present study, MBP was combined with surgery. Thus, the observed gut microbiome alterations could have resulted not only from MBP but also from the surgical procedure and the naturally changing diet during the postoperative course. Fourth, recent guidelines^44^ recommend the use of oral antibiotics alongside MBP before left-sided colorectal cancer surgery, a practice that was not implemented in the present study. It is important to note that these guidelines were not available at the time this study was conducted. However, the additional effect of oral antibiotics could have influenced the gut microbiome further. Although this aspect therefore represents a limitation of the present work, it also highlights a potential strength in focusing solely on the effects of MBP on the gut microbiome.

In conclusion, use of OP agents and RE for MBP result in similar dysbiosis. Patients who experience postoperative infections, which are the most prevalent complications after colorectal cancer surgery, exhibit distinct gut microbiome compositions on POD6, as characterised by an increased abundance of bacteria from the Actinomycetaceae family, *Actinomyces* genus, *Sutterella* uncultured species, and *Enterococcus faecalis* species. However, the study results indicate that the method chosen for MBP did not have a lasting effect on the diversity of the gut microbiome.

## Supplementary Material

znae213_Supplementary_Data

## Data Availability

This pilot two-arm RCT has been registered with ClinicalTrials.gov (NCT04013841). Raw sequencing data are publicly available in the National Center for Biotechnology Information sequence read archive (SRA) (https://www.ncbi.nlm.nih.gov/sra; SRA data accession number PRJNA1092444). Other data are available from the corresponding author upon reasonable request.
