# Supplementary material for: Impact of mechanical bowel preparation on the gut microbiome of patients undergoing left-sided colorectal cancer surgery: randomized clinical trial
Source: Br J Surg. 2024 Sep 2;111(9):znae213. doi: 10.1093/bjs/znae213 (PMC11368128; doi:10.1093/bjs/znae213)
Supplement: znae213_Supplementary_Data [file znae213_supplementary_data.docx]

**Impact of mechanical bowel preparation on the gut microbiome of patients undergoing left-sided colorectal cancer surgery****: randomized clinical trial**

Kristina Žukauskaitė^1,2^, Angela Horvath^2,3^, Žilvinas Gricius^4^, Mindaugas Kvietkauskas^4^, Bernardas Baušys^4^, Audrius Dulskas^4,5^, Justas Kuliavas^4,5^, Rimantas Baušys^5^, Simona Rūta Letautienė^5^, Ieva Vaicekauskaitė^1,5^, Rasa Sabaliauskaitė^1,5^, Augustinas Baušys^1,5,6*,^ Vanessa Stadlbauer^2,3^, Sonata Jarmalaitė^1,5^

^1^Institute of Biosciences, Life Science Center, Vilnius University, Vilnius, Lithuania;

^2^Department of Gastroenterology and Hepatology, Medical University of Graz, Graz, Austria;

^3^Center for Biomarker Research in Medicine, Graz, Austria;

^4^Clinic of Gastroenterology, Nephrourology, and Surgery, Institute of Clinical Medicine, Faculty of Medicine, Vilnius University, Vilnius, Lithuania;

^5^National Cancer Institute, Vilnius, Lithuania;

^6^Department of Pathology and Forensic Medicine, Faculty of Medicine, Institute of Biomedical Sciences, Vilnius University, Vilnius, Lithuania.

**Corresponding author:**

Augustinas Bausys MD, PhD

Department of Abdominal Surgery and Oncology

National Cancer Institute, Santariskiu str. 1, LT-08660, Vilnius, Lithuania;

Phone: +370 6 23 63 865;

E-mail: *augustinas.bausys@nvi.lt* or *augustinas.bausys@gmail.com*

ORCID ID: 000-0003-1848-2960

**Supplementary Materials - Index**

| **Supplementary Figures and Tables** |  |
| --- | --- |
| Table S1. Linear mixed-effects model results for alpha-diversity parameters. | *pag. 2* |
| Table S2. Results of PERMANOVA analysis. | *pag. 3* |
| Table S3. Culture reports of patients with postoperative infections.  Figure S1. Cladogram analysis according to the infection status. | *pag. 3-4*  *pag. 5* |
| Figure S2. The abundance of *Enterococcus faecalis* in rectal enema and oral preparation groups. | *pag. 5* |
| Figure S3. The abundance of *Enterococcus faecalis* (frequency). | *pag. 6* |
| Figure S4. The bacterial composition of the gut microbiome at the phylum level. | *pag. 6* |
| Figure S5. Redundancy analysis (RDA) of the microbiome composition. | *pag. 7* |
|  |  |
|  |  |

**Supplementary Tables and Figures**

**Table S1** Linear mixed-effects model results for alpha-diversity parameters. The table presents the estimates, standard errors (S.E.), t-values (t val.), degrees of freedom (d.f.), and p-values for four alpha diversity indices: Richness, Shannon, Inverse Simpson, and Evenness. MBP – mechanical bowel preparation. The linear mixed-effects model suggests that the factors MBP and Timepoint, as well as their interaction, do not have statistically significant effects on the assessed diversity indices, except for a marginal trend in the case of the Evenness Index interaction.

| **Source of variation** | **Est.** | **S.E.** | ***t* val.** | **d.f.** | ***p*-value** |
| --- | --- | --- | --- | --- | --- |
| *Richness index* | | | | | |
| MBP | -7.04 | 15.56 | -0.45 | 69.15 | 0.65 |
| Timepoint | -0.39 | 0.66 | -0.58 | 69.57 | 0.56 |
| MBP*Timepoint | 0.13 | 0.91 | 0.15 | 65.83 | 0.88 |
| *Shannon index* | | | | | |
| MBP | 0.00 | 0.11 | -0.04 | 76.20 | 0.97 |
| Timepoint | 0.00 | 0.00 | 0.16 | 73.57 | 0.87 |
| MBP*Timepoint | -0.01 | 0.01 | -0.80 | 69.63 | 0.43 |
| *Inverse Simpson index* | | | | | |
| MBP | -1.36 | 5.99 | -0.23 | 70.30 | 0.82 |
| Timepoint | 0.08 | 0.26 | 0.32 | 70.72 | 0.75 |
| MBP*Timepoint | -0.52 | 0.35 | -1.49 | 67.10 | 0.14 |
| *Evenness index* | | | | | |
| MBP | 0.01 | 0.01 | 0.70 | 75.99 | 0.49 |
| Timepoint | 0.00 | 0.00 | 0.81 | 73.29 | 0.42 |
| MBP*Timepoint | 0.00 | 0.00 | -1.79 | 69.31 | 0.08 |

**Table S2** Results of PERMANOVA analysis. This table presents the PERMANOVA analysis results, including the proportion of explained variation (R^2^), statistical significance (p-values), and the F value for each factor. The F value represents the ratio of between-group variation to within-group variation and assesses the significance of the factor's effect.

|  | **Source of variation** | **Sum of Squares** | **R^2^** | **F** | ***p*-value** |
| --- | --- | --- | --- | --- | --- |
| 1. | Infection | 0.654 | 0.017 | 1.675 | **0.036** |
| 2. | Timepoint | 0.327 | 0.009 | 0.839 | **0.018** |
| 3. | Infection*Timepoint | 0.258 | 0.007 | 0.662 | 0.361 |

**Table S3** Culture reports of patients with postoperative infections.

| **No.** | **MBP method** | **Infection site** | **Time of diagnosis** | **Pathogens identified in culture from the source** | **Treatment** |
| --- | --- | --- | --- | --- | --- |
| 1 | RE | Intraabdominal abscess | POD08 | *Escherichia coli*  *Proteus mirabilis*  *Enterococcus faecalis*  *Streptococcus constellatus*  *Bacteroides fragilis*  *Bacteroides thetaiotaomicron* | - Antibiot therapy with co-amoxiclav 1.2g and Metronidazole 500 mg intravenously 3 times a day from POD08 - Re-laparascopy and drainge on POD08 |
| 2 | OP | Urinary tract infections | POD02 | Not reported | - Antibiotic therapy with co-amoxiclav 1.2 g intravenously 3 times a days from POD03 |
| 3 | RE | Wound infection | POD08 | Not reported | - Wound revision on POD08 |
| 4 | RE | Urinary tract infections | POD02 | Not reported | - Antibiotic therapy with Ciprofloxacin 400 mg intravenously 2 times a days from POD02 |
| 5 | OP | Wound infection | POD06 | Not reported | - Wound revision on POD06 |
| 6 | OP | Wound infection | POD02 | *Klebsiella pneumoniae*  *Morganella morganii*  *Enterococcus faecalis* | - Wound revision on POD02 - Antibiotic therapy with co-moxiclav 1.2 g and Metronidazole 500 mg intravenously 3 times a days from POD08 |
| 7 | OP | Wound infection | POD07 | Not reported | - Wound revision on POD07 |
| 8 | RE | Intraabdominal abscess | POD07 | *Escherichia coli* | - Antibiotic therapy with co-Amoxiclav 1.2 g intravenously 3 times a days from POD06 - Ultrasound guided drainage of the abscess on POD07 |
| 9 | RE | Wound infection | POD04 | Not reported | - Wound revision on POD04 |
| 10 | RE | Intraabdominal abscess and anastomotic leakage | POD08 | *Escherichia coli*  *Klebsiella pneumoniae*  *Morganella morganii*  *Enterococcus faecium*  *Proteus mirabilis*  *Candida tropicalis* | - Antibiotic therapy with co-amoxiclav 1.2 g and Metronidazole 500 mg intravenously 3 times a days from POD04 and Linezolid 600 mg twice a day intravenously - Re-laparascopy and drainge on POD08 |
| 11 | OP | Wound infection | POD03 | Not reported | - Wound revision on POD04 |
| 12 | OP | Urinary tract infections | POD04 | Not reported | - Antibiotic therapy with Nitrofurantoin 100 mg twice a day oraly from POD04 |

*MBP – mechanical bowel preparation, RE – rectal enema, OP – oral preparation, POD – postoperative day.*


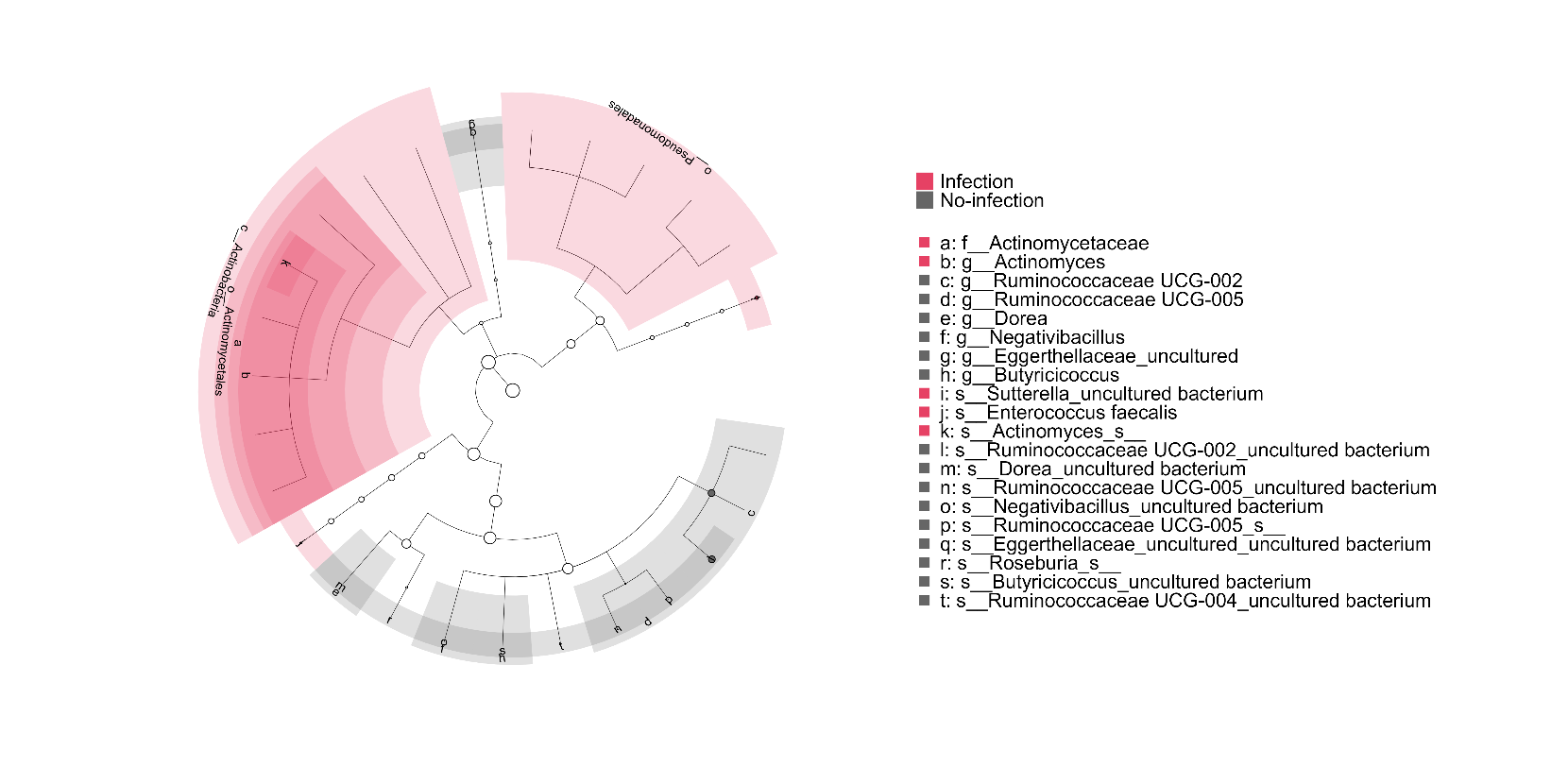


**Figure S1** Link between postoperative infection and gut microbiome changes: cladogram analysis of samples from postoperative day six in colorectal cancer patients, according to the infection status.


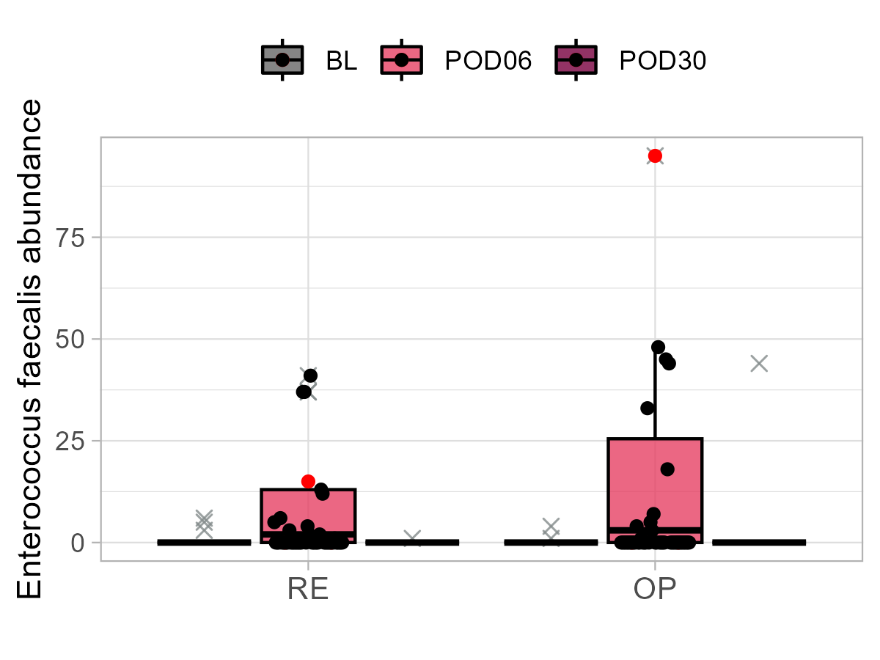


**Figure S2** The abundance of *Enterococcus faecalis* in the rectal enema and oral preparation groups across postoperative time points at day six and day 30 when compared to baseline samples. Outliers are marked in grey crosses, and those in red had blood culture reports of *E. faecalis* infection. BL – baseline, POD – postoperative day, RE – rectal enema, OP – oral preparation.


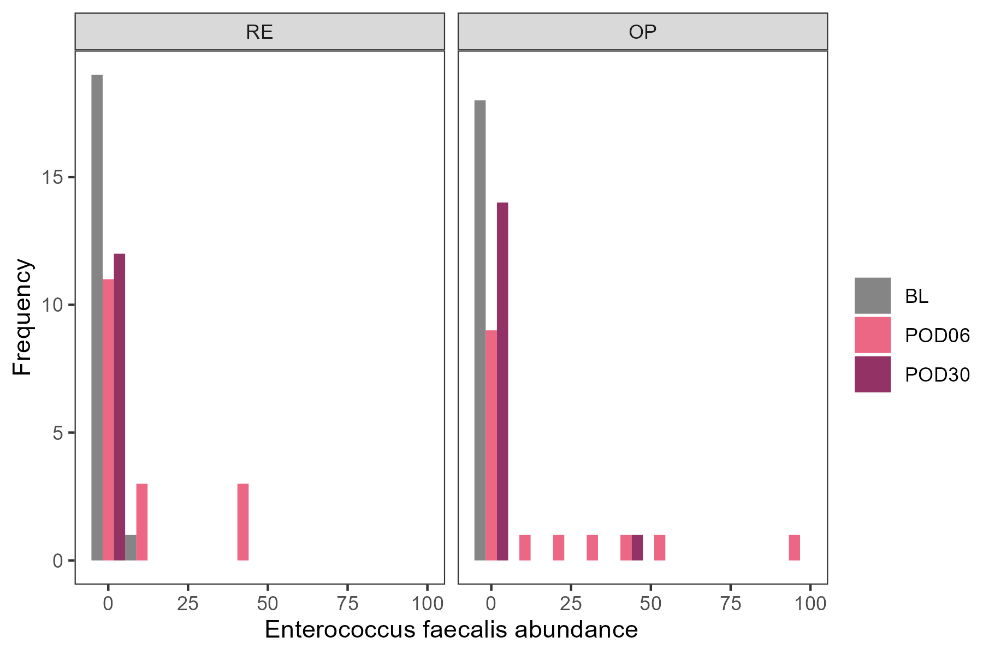


**Figure S3** The abundance of *Enterococcus faecalis* across different timepoints in colorectal cancer patients during the follow-up period according to the method of mechanical bowel preparation; RE – rectal enema, OP – oral preparation method, BL – baseline, POD – postoperative day.


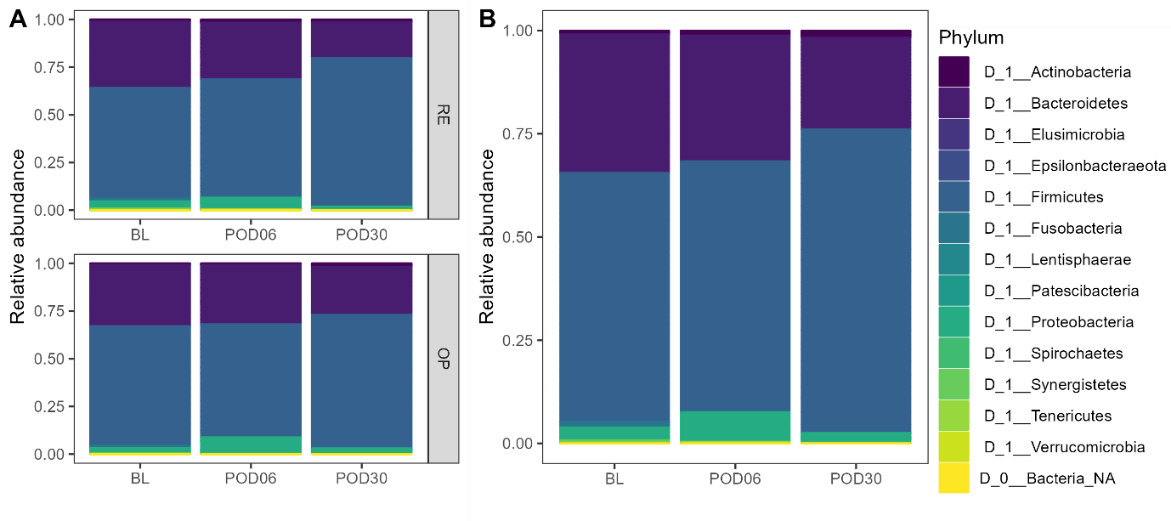


**Figure S4** Bacterial composition of the gut microbiome at phylum level in (A) different mechanical bowel prep groups and (B) all cohort at different timepoints; RE – rectal enema, OP – oral preparation method, BL – baseline, POD – postoperative day.


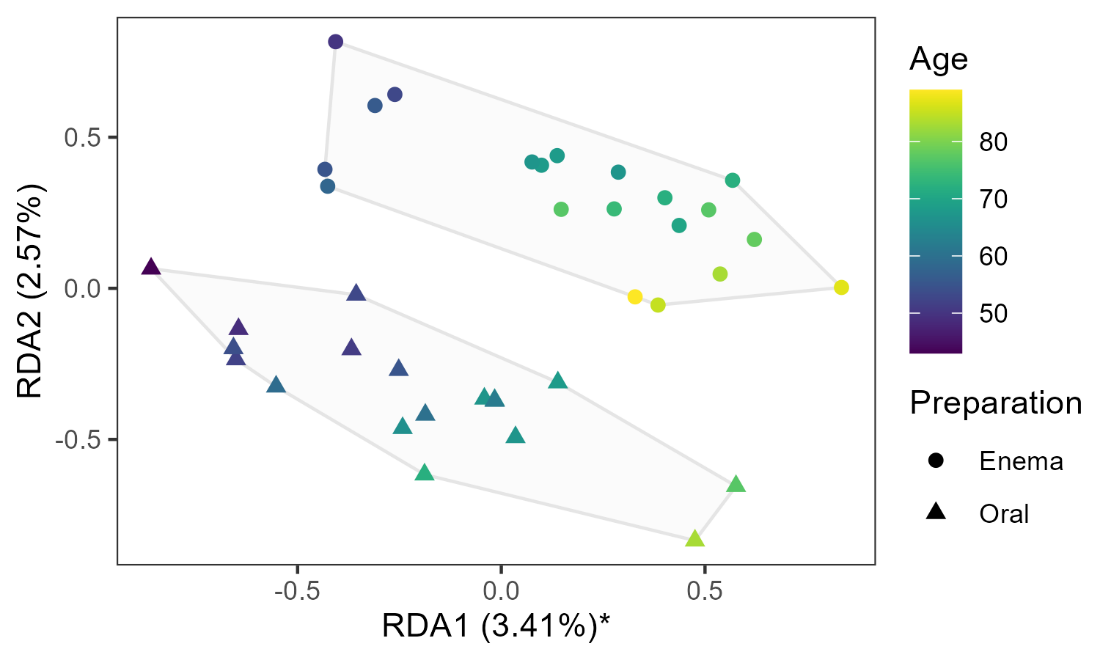


**Figure S5** Redundancy analysis (RDA) of the microbiome composition shows significant impact of age on the microbiome composition at baseline.
